# [2-Chloro-3-nitro-5-(tri­fluoro­meth­yl)phen­yl](piperidin-1-yl)methanone: structural characterization of a side product in benzo­thia­zinone synthesis

**DOI:** 10.1107/S2056989020010658

**Published:** 2020-08-11

**Authors:** Tamira Eckhardt, Richard Goddard, Ines Rudolph, Adrian Richter, Christoph Lehmann, Peter Imming, Rüdiger W. Seidel

**Affiliations:** a Institut für Pharmazie, Wolfgang-Langenbeck-Str. 4, 06120 Halle (Saale), Germany; b Max-Planck-Institut für Kohlenforschung, Kaiser-Wilhelm-Platz 1, 45470 Mülheim an der Ruhr, Germany

**Keywords:** benzo­thia­zinones, nitro­benzamides, anti-tuberculosis drugs, reaction mechanism, crystal structure

## Abstract

1,3-Benzo­thia­zin-4-ones (BTZs) are a promising new class of anti-tuberculosis drug candidates, some of which have reached clinical trials. The title compound, the benzamide derivative [2-chloro-3-nitro-5-(tri­fluoro­meth­yl)phen­yl](piper­id­in-1-yl)methanone, C_13_H_12_ClF_3_N_2_O_3_, occurs as a side product as a result of competitive reaction pathways in the nucleophilic attack during the synthesis of the BTZ 8-nitro-2-(piperidin-1-yl)-6-(tri­fluoro­meth­yl)-1,3-benzo­thia­zin-4-one, following the original synthetic route, whereby the corresponding benzoyl iso­thio­cyanate is reacted with piperidine as secondary amine. In the title compound, the nitro group and the nearly planar amide group are significantly twisted out of the plane of the benzene ring. The piperidine ring adopts a chair conformation. The tri­fluoro­methyl group exhibits slight rotational disorder with a refined ratio of occupancies of 0.972 (2):0.028 (2). There is structural evidence for inter­molecular weak C—H⋯O hydrogen bonds.

## Abstract 2

The crystal and mol­ecular structure of [2-chloro-3-nitro-5-(tri­fluoro­meth­yl)phen­yl](piperidin-1-yl)methanone, a side product in the synthesis of an 8-nitro-1,3-benzo­thia­zin-4-one, which belongs to a class of new anti-tuberculosis drug candidates, is reported.

## Chemical context   

1,3-Benzo­thia­zin-4-ones (BTZs) are promising anti-tuberculosis drug candidates, some of which have already reached clinical trials (Mikušová *et al.*, 2014 ▸; Makarov & Mikušová, 2020 ▸). Various methods for the synthesis of BTZs have been reported (Makarov *et al.*, 2007 ▸; Moellmann *et al.*, 2009 ▸; Makarov, 2011 ▸; Rudolph, 2014 ▸; Rudolph *et al.*, 2016 ▸; Zhang & Aldrich, 2019 ▸). In the original synthesis, 2-chloro­benzoyl chloride derivatives are reacted with ammonium or alkali metal thio­cyanates to form the corresponding 2-chloro­benzoyl iso­thio­cyanates (Makarov *et al.*, 2007 ▸; Moellmann *et al.*, 2009 ▸). These are reactive species and are treated *in situ* with secondary amines to afford the corresponding thio­urea deriv­atives, which undergo ring closure to give 1,3-thia­zin-4-ones *via* an intra­molecular nucleophilic substitution reaction. The latter step is favoured when electron-withdrawing substituents are present on the benzene ring.
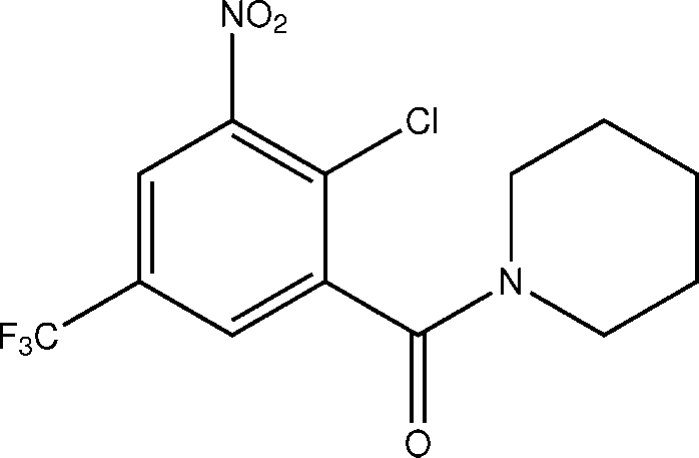


**Figure 1:**
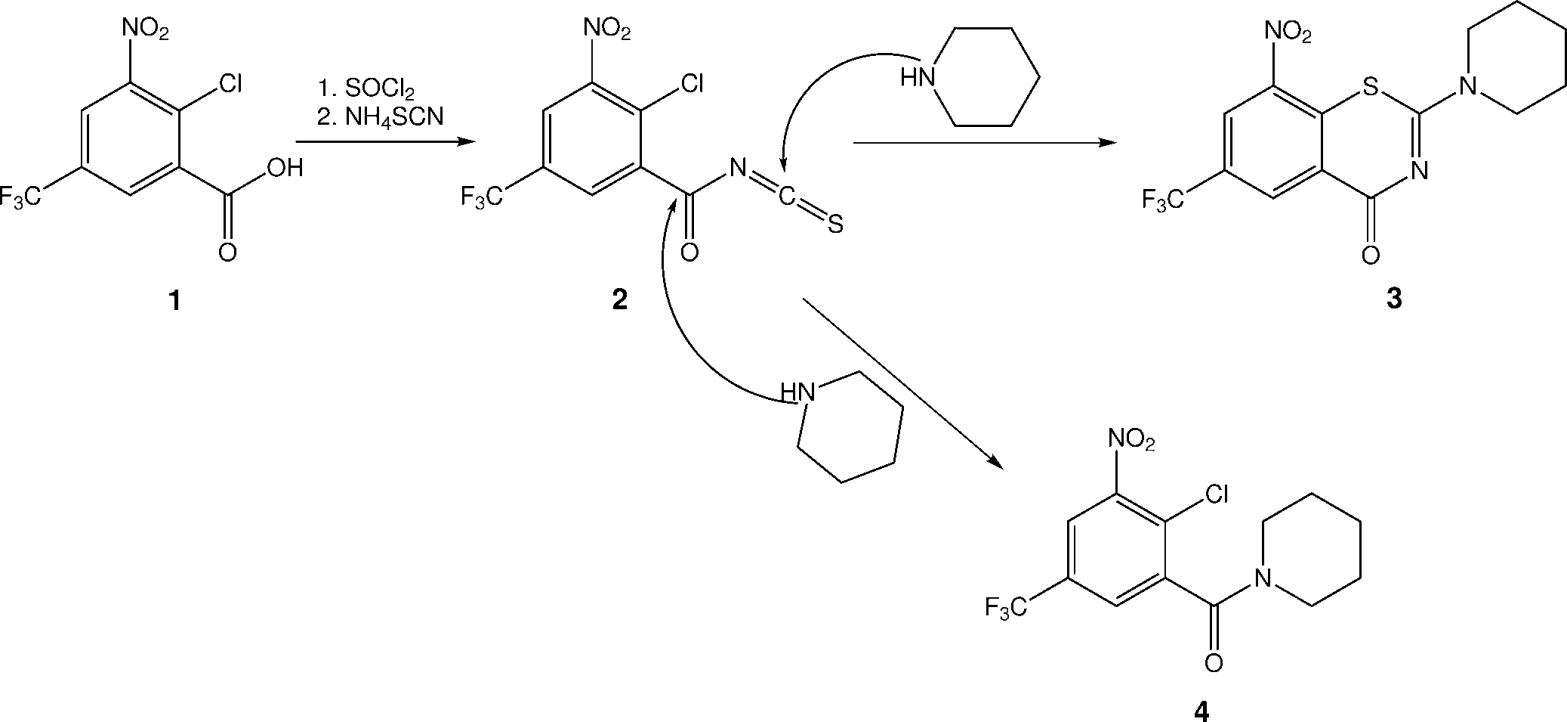
Synthetic pathway from 2-chloro-3-nitro-5-(tri­fluoro­meth­yl)benzoic acid (**1**) to BTZ **3** and side product **4**, illustrating the two different points of nucleophilic attack of piperidine at the inter­mediate 2-chloro-3-nitro-5-(tri­fluoro­meth­yl)benzoyl iso­thio­cyanate (**2**), resulting in **3** and **4** (Rudolph, 2014 ▸).

Fig. 1 ▸ depicts the synthesis following the original procedure for a BTZ previously reported by us (Rudolph, 2014 ▸; Rudolph *et al.*, 2016 ▸; Richter, Rudolph *et al.*, 2018 ▸). After treatment of 2-chloro-3-nitro-5-(tri­fluoro­meth­yl)benzoic acid (**1**) with thionyl chloride and subsequently ammonium thio­cyanate, the corresponding 2-chloro-3-nitro-5-(tri­fluoro­meth­yl)benzoyl iso­thio­cyanate (**2**) was reacted with piperidine. As illustrated, nucleophilic attack of the piperidine nitro­gen atom at the iso­thio­cyanate carbon atom leads to the anti­cipated 8-nitro-2-(piperidin-1-yl)-6-(tri­fluoro­meth­yl)-1,3-benzo­thia­zin-4-one (**3**). The alternative nucleophilic attack at the carbonyl carbon atom affords the side product (2-chloro-3-nitro-5-(tri­fluoro­meth­yl)phen­yl)(piperidin-1-yl)methanone (**4**), which was structurally characterized by X-ray crystallography in the present work. The ratio of **3** to **4** was found to vary depending on the reaction conditions. Temperatures at or below 283 K favour the formation of the anti­cipated **3**, whereas substantial amounts of **4** form at elevated temperatures (Rudolph, 2014 ▸). Since BTZs are in clinical development [see, for example, Makarov & Mikušová (2020 ▸) or Mariandyshev *et al.* (2020 ▸)], this observation is not only important for the improvement of synthetic yields but also for the compilation of known synthetic side products for drug quality control.

It is inter­esting to note that di­nitro­benzamide derivatives related to **4** have been found to have some anti-mycobacterial activity (Christophe *et al.*, 2009 ▸; Trefzer *et al.*, 2010 ▸; Tiwari *et al.*, 2013 ▸), and the non-chlorinated analogue of **4** was reported to have anti­coccidial activity (Welch *et al.*, 1969 ▸).

## Structural commentary   

**Figure 2:**
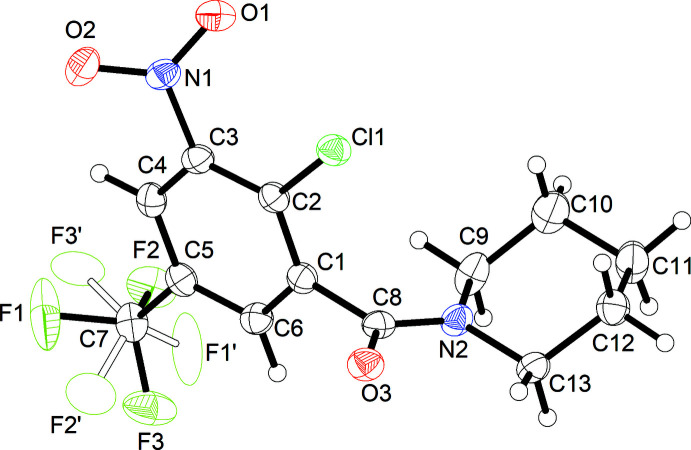
Mol­ecular structure of **4**. Displacement ellipsoids are drawn at the 50% probability level. H atoms are represented by small spheres of arbitrary radii. The minor occupancy component of the disordered tri­fluoro­methyl group is depicted by empty ellipsoids.

Fig. 2 ▸ shows the mol­ecular structure of **4** in the solid state. Selected geometric parameters are listed in Table 1 ▸. The dihedral angle between the plane of the nitro group and the mean plane of the benzene ring is 38.1 (2)°, which can be attributed to the steric demand of the neighbouring chloro substituent at the benzene ring. The tri­fluoro­methyl group exhibits rotational disorder over two sites with 97.2 (2)% occupancy for the major site. The plane of the amide group, as defined by C8, O3 and N2, is tilted out of the mean plane of the benzene ring by 79.6 (1)°. The Winkler–Dunitz parameters for the amide linkage τ (twist angle) = 1.2° and χ_N_ (pyramidalization at nitro­gen) = 4.0° indicate an almost planar amide group (Winkler & Dunitz, 1971 ▸). In the IR spectrum (see supporting information), the band at 1639 cm^−1^ can be assigned to the C=O stretching vibration of the amide group. The mol­ecule is axially chiral, although the centrosymmetric crystal structure contains both enanti­omers. The ^13^C NMR spectrum of **4** in methanol-*d*_4_ as well as chloro­form-*d* at room temperature (see supporting information) displays five distinct signals in the aliphatic region, which are assigned to the piperidine carbon atoms, indicating that the rotation about the amide C—N bond is slow in solution under these conditions. The ^13^C NMR chemical shift of the α-carbon atom C13 *syn* to the carbonyl oxygen atom of the amide group is shielded compared with that of the *anti* α-carbon atom C9. In chloro­form-*d*, the observed shielding magnitude of Δδ_C_ = 5.0 ppm is within the range expected for a benzoyl­piperidine (Rubiralta *et al.*, 1991 ▸). In the corresponding ^1^H NMR spectrum, the *syn* protons with respect to the amide carbonyl oxygen atom are deshielded compared with those in the *anti* position (Δδ_H_ = 0.58 ppm). Complete assignments of ^1^H and ^13^C NMR data in chloro­form-*d* by ^13^C,^1^H-HSQC and -HMBC NMR spectra can be found in the supporting information. Notably, the two separated methyl­ene ^1^H NMR signals assigned to C10 in chloro­form-*d* appear as one signal in methanol-*d*_4_.

In the solid state, the piperidine ring in **4** adopts a low-energy chair conformation with some minor angular deviations from ideal tetra­hedral values, resulting from planarity at N2 due to involvement in the amide linkage. The puckering parameters of the piperidine six-membered ring, as calculated with *PLATON* (Spek, 2020 ▸), are *Q* = 0.555 (2) Å, θ = 4.1 (2)° and φ = 161 (3)°. By way of comparison, the total puckering amplitude *Q* is 0.63 Å and the magnitude of distortion θ is 0° for an ideal cyclo­hexane chair (Cremer & Pople, 1975 ▸).

## Supra­molecular features   

**Figure 3:**
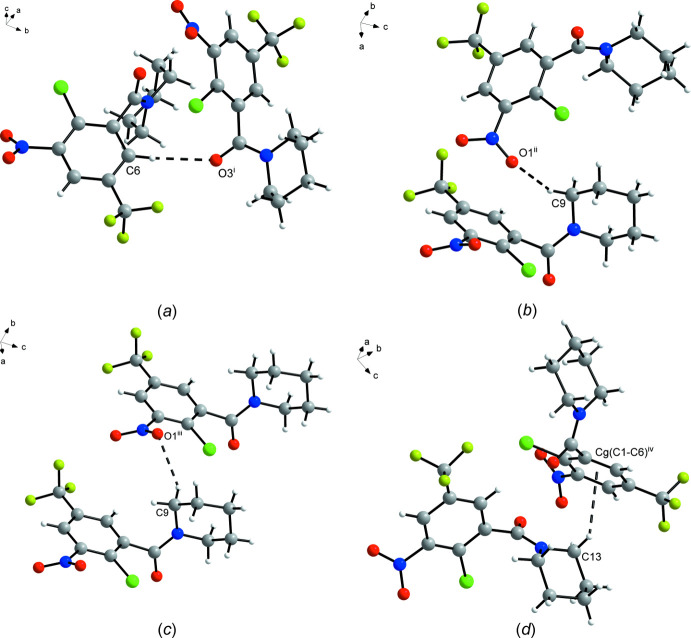
Short contacts (dashed lines) between adjacent mol­ecules in the crystal structure of **4**. The minor component of the disordered tri­fluoro­methyl group is omitted for clarity. Symmetry codes: (i) −*x* + 1, *y* + 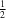, −*z* + 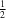; (ii) −*x* + 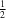, *y* + 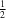, *z*; (iii) *x*, *y* + 1, *z*; (iv) *x* + 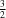, −*y* + 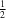, −*z*.

In general, the crystal structure of **4** appears to be dominated by close packing. According to Kitaigorodskii (1973 ▸), the space group *Pbca* is among those available for the densest packing of mol­ecules of arbitrary shape. Nevertheless, the solid-state supra­molecular structure features C—H⋯O contacts between an aromatic CH moiety and the amide oxygen atom of an adjacent mol­ecule (Fig. 3 ▸ *a*). The corres­ponding geometric parameters (Table 2 ▸) support the inter­pretation as a weak hydrogen bond (Thakuria *et al.*, 2017 ▸). These inter­actions link the mol­ecules into strands extending by 2_1_ screw symmetry in the [010] direction. The α-methyl­ene groups of the piperidine ring, on which the amide group should exert an electron-withdrawing effect, also form inter­molecular C—H⋯O and C—H⋯π contacts, respectively, to the nitro group and the benzene ring of adjacent mol­ecules (Fig. 3 ▸ *b*–*d*). The corresponding geometric parameters (Table 2 ▸), however, reveal that these contacts may not have the same significance here as the aforementioned C_aromatic_—H⋯O_amide_ short contact (Wood *et al.*, 2009 ▸). It is also worth noting that π–π stacking of the aromatic rings is not observed.

## Database survey   

A search of the Cambridge Structural Database (CSD; version 5.41 with March 2020 updates; Groom *et al.*, 2016 ▸) for related substituted *N*-benzoyl-piperidine compounds revealed about 30 structures, of which (2-chloro-3,5-di­nitro­phen­yl)(piperidin-1-yl)methanone (CSD refcode: URALIJ; Luo *et al.*, 2011 ▸) is structurally most related to **4**. Similar to **4**, the 3-nitro group with the neighbouring chloro substituent is tilted out of the mean plane of the benzene ring by 36.2°. At 75.8°, the dihedral angle between the amide plane and the mean plane of the benzene ring is comparable with that in **4**. Likewise, the piperidine ring exhibits a chair conformation with a planar structure at the nitro­gen atom. In contrast to **4**, the solid-state supra­molecular structure of URALIJ exhibits π–π stacking of the aromatic rings. Inter­estingly, a CSD search for the 2-chloro-3-nitro-5-(tri­fluoro­meth­yl)phenyl moiety present in **4** led to only one structure, *viz.* 2-chloro-1,3-di­nitro-5-(tri­fluoro­meth­yl)benzene (JIHNUM; del Casino *et al.*, 2018 ▸), also known as chloralin, which is active against *Plasmodium*, but which also shows toxicity in mice.

## Anti-mycobacterial evaluation   

The anti-mycobacterial activity of **4** was evaluated against *Mycobacterium smegmatis* mc^2^ 155 and *Mycobacterium abscessus* ATCC19977, using broth microdilution assays [for the assay protocols, see the supporting information and Richter, Strauch *et al.* (2018 ▸)]. For both mycobacterial species, no growth inhibition was detectable up to a concentration of 100 µM. For *M. smegmatis*, the findings are consistent with the activity data for a related nitro­benzamide derivative reported by Tiwari *et al.* (2013 ▸). CT319, a 3-nitro-5-(tri­fluoro­meth­yl)benzamide derivative, however, showed activity against *M. smegmatis* mc^2^ 155 and other mycobacterial strains (Trefzer *et al.*, 2010 ▸).

## Synthesis and crystallization   

Chemicals were purchased and used as received. The synthesis of **1** is described elsewhere (Welch *et al.*, 1969 ▸). Solvents were of reagent grade and were distilled before use. The IR spectrum was measured on a Bruker TENSOR II FT–IR spectrometer at a resolution of 4 cm^−1^. NMR spectra were recorded at room temperature on an Agilent Technologies VNMRS 400 MHz NMR spectrometer (abbreviations: *d* = doublet, *q* = quartet, *m* = multiplet). Chemical shifts are referenced to the residual signals of methanol-*d*_4_ (δ_H_ = 3.35 ppm, δ_C_ = 49.3 ppm) or chloro­form-*d* (δ_H_ = 7.26 ppm, δ_C_ = 77.2 ppm).

2.7 mL (37.0 mmol) of SOCl_2_ were added to a stirred solution of **1** (5.00 g,18.5 mmol) in toluene, and the mixture was heated to reflux for two h. The solvent was subsequently removed under reduced pressure, and the acid chloride thus obtained was used without purification. The residue was taken up in 6.5 mL of aceto­nitrile and a solution of 1.41 g (18.5 mmol) NH_4_SCN in 55 mL of aceto­nitrile was added dropwise with stirring to obtain **2** *in situ*. After stirring for 5 min at 313 K, the resulting NH_4_Cl precipitate was filtered off, and 3.7 mL (37.0 mmol) of piperidine were added. The mixture was refluxed overnight, and then the solvent was removed under reduced pressure. Water was added to the residue and, after extraction with di­chloro­methane, the organic phase was washed with 10% aqueous NaHCO_3_ and dried over MgSO_4_. After removal of the solvent, the crude product was subjected to flash chromatography on silica gel, eluting with ethyl acetate/*n*-heptane (gradient 10–50% *v*/*v*), to isolate 1.09 g (3.0 mmol, 16%) of **3** and a minor amount of the side product **4**. ^1^H and ^13^C NMR spectroscopic and mass spectrometric data of **3** were in agreement with those in the literature (Rudolph, 2014 ▸; Rudolph *et al.*, 2016 ▸). Crystals of **4** suitable for X-ray crystallography were obtained from a solution in ethyl acetate/heptane (1:1) by slow evaporation of the solvents at room temperature. NMR spectroscopic data for **4**:

^1^H NMR (400 MHz, CD_3_OD) δ 8.42 (*d*, ^4^ *J*_meta_ = 2.2 Hz, 1H, Ar—*H*), 8.09 (*d*, ^4^ *J*_meta_ = 2.2 Hz, 1H, Ar—*H*), 3.88–3.71 (*m*, 2H, N—C*H*_2_), 3.33–3.21 (*m*, 2H, N—C*H*_2_), 1.76 (*m*, 4H, CH_2_), 1.64 (*m*, 2H, CH_2_) ppm; ^13^C NMR (101 MHz, CD_3_OD) δ 165.5, 150.7, 141.8, 132.3 (*q*, ^2^ *J*_C,F_ = 35 Hz), 129.2 (*q*, ^3^ *J*_C,F_ = 4 Hz), 128.1, 124.4 (*q*, ^3^ *J*_C,F_ = 4 Hz), 124.1 (*q*, ^1^ *J*_C,F_ = 273 Hz), 49.5, 44.3, 27.6, 26.7, 25.5 ppm.

^1^H NMR (400 MHz, CDCl_3_ δ) 8.07 (*d*, ^4^ *J*_meta_ = 2.0 Hz, 1H, C4—*H*), 7.73 (*d*, ^4^ *J*_meta_ = 2.0 Hz, 1H, C6—*H*), 3.83–3.68 (*m*, 2H, C13—C*H*_2_), 3.22 (*ddd*, ^2^ *J*_gem_ = 13.2 Hz, ^3^ *J*_vic_ = 7.1, 4.0 Hz, 1H, C9—C*H*_2_), 3.15 (*ddd*, ^2^ *J*_gem_ = 13.2 Hz, ^3^ *J*_vic_ = 7.1, 4.0 Hz, 1H, C9—C*H*_2_), 1.70 (*m*, 4H, C11, C12—C*H*_2_), 1.65–1.57 (*m*, 1H, C10—C*H*_2_), 1.56–1.47 (*m*, 1H, C10—C*H*_2_) ppm; ^13^C NMR (101 MHz, CDCl_3_ δ) 163.5 (C8, C=O), 148.9 (C3), 141.1 (C1), 131.2 (*q*, ^2^ *J*_C,F_ = 35 Hz, C5), 127.8 (*q*, ^3^ *J*_C,F_ = 4 Hz, C6), 127.6 (C2), 122.7 (*q*, ^3^ *J*_C,F_ = 4 Hz, C4), 122.4 (*q*, ^1^ *J*_C,F_ = 273 Hz, C7), 48.3 (C9), 43.3 (C13), 26.7 (C10), 25.7 (C12), 24.6 (C11) ppm.

## Refinement   

Crystal data, data collection and structure refinement details are summarized in Table 3 ▸. The rotational disorder of the tri­fluoro­methyl group was refined using a split model with similar distance restraints on the 1,2- and 1,3-distances and equal atomic displacement parameters for opposite fluorine atoms belonging to different disorder sites. Refinement of the ratio of occupancies by means of a free variable resulted in 0.972 (2):0.028 (2). Hydrogen-atom positions were calculated geometrically with C_a_—H = 0.95 Å and C_m_—H = 0.99 Å (a = aromatic and m = methyl­ene), and refined with the appropriate riding model and *U*_iso_(H) = 1.2 *U*_eq_(C).

## Supplementary Material

Crystal structure: contains datablock(s) global, I. DOI: 10.1107/S2056989020010658/vm2238sup1.cif

Structure factors: contains datablock(s) I. DOI: 10.1107/S2056989020010658/vm2238Isup2.hkl

Supporting information file. DOI: 10.1107/S2056989020010658/vm2238Isup3.cdx

IR and NMR spectra, assay protocol for anti-mycobacterial activity. DOI: 10.1107/S2056989020010658/vm2238sup4.pdf

Supporting information file. DOI: 10.1107/S2056989020010658/vm2238Isup5.cml

CCDC reference: 2021003

Additional supporting information:  crystallographic information; 3D view; checkCIF report

## Figures and Tables

**Table 1:** Selected geometric parameters (Å, °)

C1—C8	1.510 (3)	C7—F3	1.328 (3)
C2—Cl1	1.725 (2)	C7—F2	1.336 (3)
C3—N1	1.468 (3)	C8—O3	1.234 (2)
C5—C7	1.497 (3)	C8—N2	1.342 (3)
C7—F1	1.325 (3)		
			
C4—C3—N1	116.41 (17)	N2—C9—C10	110.59 (18)
C2—C3—N1	122.35 (18)	C9—C10—C11	110.61 (19)
F1—C7—F3	107.69 (19)	C12—C11—C10	109.74 (18)
F1—C7—F2	105.98 (19)	C13—C12—C11	111.01 (18)
F3—C7—F2	105.59 (17)	N2—C13—C12	111.35 (17)
F1—C7—C5	112.43 (17)	O2—N1—O1	124.48 (17)
F3—C7—C5	112.80 (18)	O2—N1—C3	117.04 (17)
F2—C7—C5	111.86 (17)	O1—N1—C3	118.44 (16)
O3—C8—N2	124.72 (18)	C8—N2—C13	120.26 (16)
O3—C8—C1	118.43 (18)	C8—N2—C9	124.74 (16)
N2—C8—C1	116.85 (17)	C13—N2—C9	114.89 (16)
			
C4—C3—N1—O2	36.6 (2)	O3—C8—N2—C13	3.0 (3)
C2—C3—N1—O2	−143.29 (19)	C1—C8—N2—C13	−176.62 (17)
C4—C3—N1—O1	−141.34 (18)	O3—C8—N2—C9	179.0 (2)
C2—C3—N1—O1	38.8 (3)	C1—C8—N2—C9	−0.6 (3)

**Table 2:** Hydrogen-bond geometry (Å, °)

*D*—H⋯*A*	*D*—H	H⋯*A*	*D*⋯*A*	*D*—H⋯*A*
C6—H6⋯O3^i^	0.95	2.59	3.526 (3)	169
C9—H9*A*⋯O1^ii^	0.99	2.45	3.361 (3)	154
C9—H9*B*⋯O1^iii^	0.99	2.58	3.369 (3)	137
C13—H13*A*⋯*Cg*(C1–C6)^iv^	0.99	2.92	3.447 (2)	114

Symmetry codes: (i) 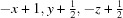; (ii) 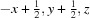; (iii) 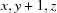; (iv) 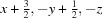.

**Table 3:** Experimental details

Crystal data	
Chemical formula	C_13_H_12_ClF_3_N_2_O_3_
*M*_r_	336.70
Crystal system, space group	Orthorhombic, *P* *b* *c* *a*
Temperature (K)	100
*a*, *b*, *c* (Å)	18.0904 (7), 7.8971 (3), 19.8043 (8)
*V* (Å^3^)	2829.28 (19)
*Z*	8
Radiation type	Cu *K*α
μ (mm^−1^)	2.88
Crystal size (mm)	0.59 × 0.50 × 0.44

Data collection	
Diffractometer	Bruker Kappa Mach3 APEXII
Absorption correction	Gaussian (*SADABS*; Krause *et al.*, 2015 ▸)
*T*_min_, *T*_max_	0.297, 0.586
No. of measured, independent and observed [*I* > 2σ(*I*)] reflections	49954, 2784, 2699
*R*_int_	0.041
(sin θ/λ)_max_ (Å^−1^)	0.617

Refinement	
*R*[*F* ^2^ > 2σ(*F* ^2^)], *wR*(*F* ^2^), *S*	0.043, 0.115, 1.15
No. of reflections	2784
No. of parameters	209
No. of restraints	45
H-atom treatment	H-atom parameters constrained
Δρ_max_, Δρ_min_ (e Å^−3^)	0.32, −0.32

Computer programs: *APEX3* (Bruker, 2017 ▸), *SAINT* (Bruker, 2004 ▸), *SHELXT2014/4* (Sheldrick, 2015*a* ▸), *SHELXL2018/3* (Sheldrick, 2015*b* ▸), *DIAMOND* (Brandenburg, 2018 ▸), *enCIFer* (Allen *et al.*, 2004 ▸) and *publCIF* (Westrip, 2010 ▸).

## supplementary crystallographic information

### Crystal data

**Table:**

C_13_H_12_ClF_3_N_2_O_3_	*D*_x_ = 1.581 Mg m^−^^3^
*M**_r_* = 336.70	Cu *K*α radiation, λ = 1.54178 Å
Orthorhombic, *P**b**c**a*	Cell parameters from 9968 reflections
*a* = 18.0904 (7) Å	θ = 4.9–71.6°
*b* = 7.8971 (3) Å	µ = 2.88 mm^−^^1^
*c* = 19.8043 (8) Å	*T* = 100 K
*V* = 2829.28 (19) Å^3^	Block, colourless
*Z* = 8	0.59 × 0.50 × 0.44 mm
*F*(000) = 1376	

### Data collection

**Table:**

Bruker Kappa Mach3 APEXII diffractometer	2784 independent reflections
Radiation source: 0.2 × 2 mm^2^ focus rotating anode	2699 reflections with *I* > 2σ(*I*)
MONTEL graded multilayer optics monochromator	*R*_int_ = 0.041
Detector resolution: 66.67 pixels mm^-1^	θ_max_ = 72.2°, θ_min_ = 4.9°
φ– and ω–scans	*h* = −21→22
Absorption correction: gaussian (SADABS; Krause *et al.*, 2015)	*k* = −9→9
*T*_min_ = 0.297, *T*_max_ = 0.586	*l* = −24→24
49954 measured reflections	

### Refinement

**Table:**

Refinement on *F*^2^	Primary atom site location: dual
Least-squares matrix: full	Secondary atom site location: difference Fourier map
*R*[*F*^2^ > 2σ(*F*^2^)] = 0.043	Hydrogen site location: inferred from neighbouring sites
*wR*(*F*^2^) = 0.115	H-atom parameters constrained
*S* = 1.15	*w* = 1/[σ^2^(*F*_o_^2^) + (0.0508*P*)^2^ + 2.7994*P*] where *P* = (*F*_o_^2^ + 2*F*_c_^2^)/3
2784 reflections	(Δ/σ)_max_ < 0.001
209 parameters	Δρ_max_ = 0.32 e Å^−^^3^
45 restraints	Δρ_min_ = −0.32 e Å^−^^3^

### Special details

**Table:**

Geometry. All esds (except the esd in the dihedral angle between two l.s. planes) are estimated using the full covariance matrix. The cell esds are taken into account individually in the estimation of esds in distances, angles and torsion angles; correlations between esds in cell parameters are only used when they are defined by crystal symmetry. An approximate (isotropic) treatment of cell esds is used for estimating esds involving l.s. planes.

### Fractional atomic coordinates and isotropic or equivalent isotropic displacement parameters (Å^2^)

**Table:**

	*x*	*y*	*z*	*U*_iso_*/*U*_eq_	Occ. (<1)
C1	0.41556 (10)	0.3250 (3)	0.23581 (10)	0.0230 (4)	
C2	0.38370 (10)	0.1651 (3)	0.23833 (10)	0.0224 (4)	
C3	0.35007 (10)	0.1004 (3)	0.18023 (10)	0.0227 (4)	
C4	0.34763 (10)	0.1927 (3)	0.12111 (10)	0.0243 (4)	
H4	0.325215	0.146458	0.081847	0.029*	
C5	0.37831 (10)	0.3541 (3)	0.11961 (10)	0.0242 (4)	
C6	0.41190 (11)	0.4206 (3)	0.17666 (10)	0.0250 (4)	
H6	0.432417	0.531321	0.175487	0.030*	
C7	0.37425 (12)	0.4549 (3)	0.05572 (11)	0.0298 (5)	
C8	0.45848 (10)	0.3906 (2)	0.29575 (10)	0.0232 (4)	
C9	0.34929 (13)	0.5683 (3)	0.32422 (11)	0.0327 (5)	
H9A	0.325497	0.503425	0.287381	0.039*	
H9B	0.351745	0.688568	0.310277	0.039*	
C10	0.30335 (12)	0.5528 (3)	0.38813 (13)	0.0404 (6)	
H10A	0.296350	0.431647	0.399304	0.048*	
H10B	0.254021	0.603627	0.380559	0.048*	
C11	0.34134 (13)	0.6421 (3)	0.44689 (11)	0.0373 (5)	
H11A	0.344826	0.764952	0.437412	0.045*	
H11B	0.311898	0.626777	0.488581	0.045*	
C12	0.41843 (12)	0.5691 (3)	0.45695 (10)	0.0294 (5)	
H12A	0.443955	0.632047	0.493365	0.035*	
H12B	0.414464	0.449164	0.470978	0.035*	
C13	0.46343 (11)	0.5807 (3)	0.39245 (10)	0.0277 (4)	
H13A	0.473754	0.701122	0.382205	0.033*	
H13B	0.511333	0.522352	0.398982	0.033*	
N1	0.31581 (9)	−0.0681 (2)	0.17845 (9)	0.0253 (4)	
N2	0.42423 (9)	0.5038 (2)	0.33544 (8)	0.0249 (4)	
O1	0.28104 (8)	−0.1161 (2)	0.22819 (8)	0.0324 (4)	
O2	0.32229 (8)	−0.1493 (2)	0.12611 (8)	0.0341 (4)	
O3	0.52198 (7)	0.33806 (19)	0.30471 (7)	0.0288 (3)	
F1	0.38438 (14)	0.3603 (2)	0.00122 (7)	0.0711 (7)	0.972 (2)
F2	0.30831 (8)	0.52847 (19)	0.04797 (8)	0.0418 (4)	0.972 (2)
F3	0.42321 (8)	0.5801 (2)	0.05379 (8)	0.0470 (4)	0.972 (2)
F1'	0.352 (3)	0.612 (3)	0.0688 (18)	0.0711 (7)	0.028 (2)
F2'	0.4387 (11)	0.472 (6)	0.0269 (18)	0.0418 (4)	0.028 (2)
F3'	0.325 (2)	0.397 (5)	0.0129 (15)	0.0470 (4)	0.028 (2)
Cl1	0.38924 (3)	0.05417 (6)	0.31320 (2)	0.02635 (16)	

### Atomic displacement parameters (Å^2^)

**Table:**

	*U*^11^	*U*^22^	*U*^33^	*U*^12^	*U*^13^	*U*^23^
C1	0.0194 (9)	0.0250 (9)	0.0246 (9)	0.0021 (7)	0.0012 (7)	−0.0019 (8)
C2	0.0186 (8)	0.0246 (9)	0.0239 (9)	0.0021 (7)	0.0011 (7)	0.0002 (8)
C3	0.0166 (8)	0.0219 (9)	0.0296 (10)	0.0011 (7)	0.0009 (7)	−0.0021 (8)
C4	0.0202 (9)	0.0288 (10)	0.0239 (9)	0.0050 (8)	−0.0004 (7)	−0.0031 (8)
C5	0.0219 (9)	0.0261 (10)	0.0247 (10)	0.0056 (8)	0.0021 (7)	0.0011 (8)
C6	0.0248 (10)	0.0226 (9)	0.0277 (10)	0.0006 (8)	0.0004 (8)	0.0002 (8)
C7	0.0350 (11)	0.0292 (11)	0.0251 (10)	0.0056 (9)	0.0005 (8)	−0.0002 (8)
C8	0.0225 (9)	0.0226 (9)	0.0245 (9)	−0.0035 (8)	−0.0003 (7)	0.0033 (8)
C9	0.0335 (11)	0.0298 (11)	0.0347 (11)	0.0094 (9)	−0.0117 (9)	−0.0088 (9)
C10	0.0200 (10)	0.0517 (15)	0.0495 (14)	0.0058 (9)	−0.0038 (9)	−0.0188 (11)
C11	0.0324 (11)	0.0474 (14)	0.0322 (11)	0.0039 (10)	−0.0011 (9)	−0.0114 (10)
C12	0.0308 (11)	0.0327 (11)	0.0247 (10)	−0.0010 (9)	−0.0013 (8)	0.0002 (8)
C13	0.0267 (10)	0.0306 (10)	0.0257 (10)	−0.0070 (8)	−0.0028 (8)	−0.0018 (8)
N1	0.0190 (8)	0.0260 (9)	0.0310 (9)	−0.0009 (6)	−0.0037 (7)	−0.0016 (7)
N2	0.0213 (8)	0.0277 (9)	0.0257 (8)	−0.0010 (7)	−0.0042 (7)	−0.0030 (7)
O1	0.0267 (7)	0.0334 (8)	0.0371 (8)	−0.0062 (6)	0.0010 (6)	0.0028 (7)
O2	0.0348 (8)	0.0320 (8)	0.0356 (8)	−0.0010 (6)	−0.0045 (6)	−0.0098 (7)
O3	0.0204 (7)	0.0324 (8)	0.0337 (8)	0.0005 (6)	−0.0017 (6)	−0.0003 (6)
F1	0.151 (2)	0.0382 (9)	0.0239 (7)	0.0278 (10)	0.0154 (9)	−0.0002 (6)
F2	0.0336 (7)	0.0457 (8)	0.0460 (8)	0.0053 (6)	−0.0056 (6)	0.0175 (7)
F3	0.0389 (8)	0.0567 (10)	0.0453 (8)	−0.0142 (7)	−0.0052 (6)	0.0251 (7)
F1'	0.151 (2)	0.0382 (9)	0.0239 (7)	0.0278 (10)	0.0154 (9)	−0.0002 (6)
F2'	0.0336 (7)	0.0457 (8)	0.0460 (8)	0.0053 (6)	−0.0056 (6)	0.0175 (7)
F3'	0.0389 (8)	0.0567 (10)	0.0453 (8)	−0.0142 (7)	−0.0052 (6)	0.0251 (7)
Cl1	0.0267 (3)	0.0273 (3)	0.0250 (3)	−0.00271 (18)	0.00002 (17)	0.00364 (17)

### Geometric parameters (Å, º)

**Table:**

C1—C2	1.389 (3)	C8—N2	1.342 (3)
C1—C6	1.395 (3)	C9—N2	1.465 (3)
C1—C8	1.510 (3)	C9—C10	1.519 (3)
C2—C3	1.398 (3)	C9—H9A	0.9900
C2—Cl1	1.725 (2)	C9—H9B	0.9900
C3—C4	1.380 (3)	C10—C11	1.525 (3)
C3—N1	1.468 (3)	C10—H10A	0.9900
C4—C5	1.390 (3)	C10—H10B	0.9900
C4—H4	0.9500	C11—C12	1.522 (3)
C5—C6	1.386 (3)	C11—H11A	0.9900
C5—C7	1.497 (3)	C11—H11B	0.9900
C6—H6	0.9500	C12—C13	1.518 (3)
C7—F2'	1.304 (14)	C12—H12A	0.9900
C7—F3'	1.314 (14)	C12—H12B	0.9900
C7—F1	1.325 (3)	C13—N2	1.465 (2)
C7—F3	1.328 (3)	C13—H13A	0.9900
C7—F1'	1.332 (14)	C13—H13B	0.9900
C7—F2	1.336 (3)	N1—O2	1.225 (2)
C8—O3	1.234 (2)	N1—O1	1.229 (2)
			
C2—C1—C6	120.16 (18)	C10—C9—H9A	109.5
C2—C1—C8	119.82 (17)	N2—C9—H9B	109.5
C6—C1—C8	119.90 (17)	C10—C9—H9B	109.5
C1—C2—C3	118.92 (18)	H9A—C9—H9B	108.1
C1—C2—Cl1	117.93 (15)	C9—C10—C11	110.61 (19)
C3—C2—Cl1	123.13 (16)	C9—C10—H10A	109.5
C4—C3—C2	121.24 (18)	C11—C10—H10A	109.5
C4—C3—N1	116.41 (17)	C9—C10—H10B	109.5
C2—C3—N1	122.35 (18)	C11—C10—H10B	109.5
C3—C4—C5	119.32 (18)	H10A—C10—H10B	108.1
C3—C4—H4	120.3	C12—C11—C10	109.74 (18)
C5—C4—H4	120.3	C12—C11—H11A	109.7
C6—C5—C4	120.33 (18)	C10—C11—H11A	109.7
C6—C5—C7	120.58 (19)	C12—C11—H11B	109.7
C4—C5—C7	119.09 (18)	C10—C11—H11B	109.7
C5—C6—C1	119.98 (19)	H11A—C11—H11B	108.2
C5—C6—H6	120.0	C13—C12—C11	111.01 (18)
C1—C6—H6	120.0	C13—C12—H12A	109.4
F2'—C7—F3'	111.1 (19)	C11—C12—H12A	109.4
F1—C7—F3	107.69 (19)	C13—C12—H12B	109.4
F2'—C7—F1'	105.2 (19)	C11—C12—H12B	109.4
F3'—C7—F1'	104.0 (19)	H12A—C12—H12B	108.0
F1—C7—F2	105.98 (19)	N2—C13—C12	111.35 (17)
F3—C7—F2	105.59 (17)	N2—C13—H13A	109.4
F2'—C7—C5	112.3 (15)	C12—C13—H13A	109.4
F3'—C7—C5	113.2 (15)	N2—C13—H13B	109.4
F1—C7—C5	112.43 (17)	C12—C13—H13B	109.4
F3—C7—C5	112.80 (18)	H13A—C13—H13B	108.0
F1'—C7—C5	110.3 (15)	O2—N1—O1	124.48 (17)
F2—C7—C5	111.86 (17)	O2—N1—C3	117.04 (17)
O3—C8—N2	124.72 (18)	O1—N1—C3	118.44 (16)
O3—C8—C1	118.43 (18)	C8—N2—C13	120.26 (16)
N2—C8—C1	116.85 (17)	C8—N2—C9	124.74 (16)
N2—C9—C10	110.59 (18)	C13—N2—C9	114.89 (16)
N2—C9—H9A	109.5		
			
C6—C1—C2—C3	1.9 (3)	C6—C5—C7—F1'	−47 (3)
C8—C1—C2—C3	−174.16 (17)	C4—C5—C7—F1'	133 (3)
C6—C1—C2—Cl1	−179.32 (15)	C6—C5—C7—F2	−99.4 (2)
C8—C1—C2—Cl1	4.6 (2)	C4—C5—C7—F2	80.3 (2)
C1—C2—C3—C4	−0.5 (3)	C2—C1—C8—O3	77.8 (2)
Cl1—C2—C3—C4	−179.22 (14)	C6—C1—C8—O3	−98.3 (2)
C1—C2—C3—N1	179.37 (16)	C2—C1—C8—N2	−102.6 (2)
Cl1—C2—C3—N1	0.6 (3)	C6—C1—C8—N2	81.4 (2)
C2—C3—C4—C5	−0.9 (3)	N2—C9—C10—C11	55.5 (3)
N1—C3—C4—C5	179.25 (16)	C9—C10—C11—C12	−56.9 (3)
C3—C4—C5—C6	0.9 (3)	C10—C11—C12—C13	55.8 (3)
C3—C4—C5—C7	−178.87 (18)	C11—C12—C13—N2	−53.4 (2)
C4—C5—C6—C1	0.5 (3)	C4—C3—N1—O2	36.6 (2)
C7—C5—C6—C1	−179.75 (18)	C2—C3—N1—O2	−143.29 (19)
C2—C1—C6—C5	−1.9 (3)	C4—C3—N1—O1	−141.34 (18)
C8—C1—C6—C5	174.13 (18)	C2—C3—N1—O1	38.8 (3)
C6—C5—C7—F2'	70 (2)	O3—C8—N2—C13	3.0 (3)
C4—C5—C7—F2'	−110 (2)	C1—C8—N2—C13	−176.62 (17)
C6—C5—C7—F3'	−163 (2)	O3—C8—N2—C9	179.0 (2)
C4—C5—C7—F3'	17 (2)	C1—C8—N2—C9	−0.6 (3)
C6—C5—C7—F1	141.5 (2)	C12—C13—N2—C8	−130.0 (2)
C4—C5—C7—F1	−38.8 (3)	C12—C13—N2—C9	53.6 (2)
C6—C5—C7—F3	19.5 (3)	C10—C9—N2—C8	129.2 (2)
C4—C5—C7—F3	−160.82 (18)	C10—C9—N2—C13	−54.6 (2)

### Hydrogen-bond geometry (Å, º)

**Table:**

*D*—H···*A*	*D*—H	H···*A*	*D*···*A*	*D*—H···*A*
C6—H6···O3^i^	0.95	2.59	3.526 (3)	169
C9—H9*A*···O1^ii^	0.99	2.45	3.361 (3)	154
C9—H9*B*···O1^iii^	0.99	2.58	3.369 (3)	137
C13—H13*A*···*Cg*(C1–C6)^iv^	0.99	2.92	3.447 (2)	114

Symmetry codes: (i) −*x*+1, *y*+1/2, −*z*+1/2; (ii) −*x*+1/2, *y*+1/2, *z*; (iii) *x*, *y*+1, *z*; (iv) *x*+3/2, −*y*+1/2, −*z*.
