# Supplementary material for: [2-Chloro-3-nitro-5-(tri­fluoro­meth­yl)phen­yl](piperidin-1-yl)methanone: structural characterization of a side product in benzo­thia­zinone synthesis
Source: Acta Crystallogr E Crystallogr Commun. 2020 Aug 11;76(Pt 9):1442–6. doi: 10.1107/S2056989020010658 (PMC7472761; doi:10.1107/S2056989020010658)
Supplement: Supplementary file 4 [file e-76-01442-sup4.pdf]

Supporting information for

(2-Chloro-3-nitro-5-(trifluoromethyl)phenyl)(piperidin-1-yl)methanone: structural characterization of a side product in benzothiazinone synthesis

T. Eckhardt, R. Goddard, I. Rudolph, A. Richter, C. Lehmann, P. Imming and R. W. Seidel

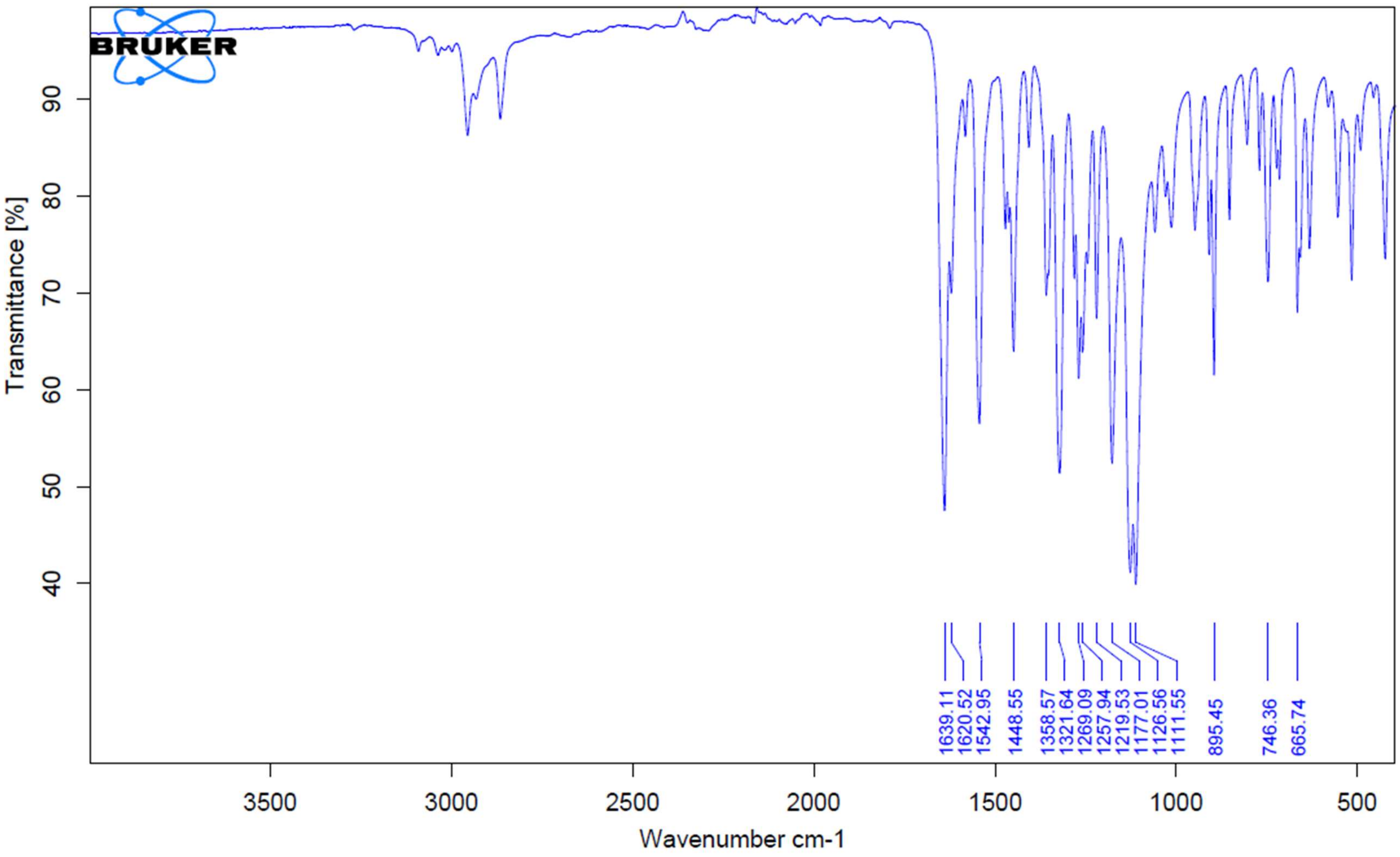

Figure S1 ATR FT-IR spectrum of **4**.

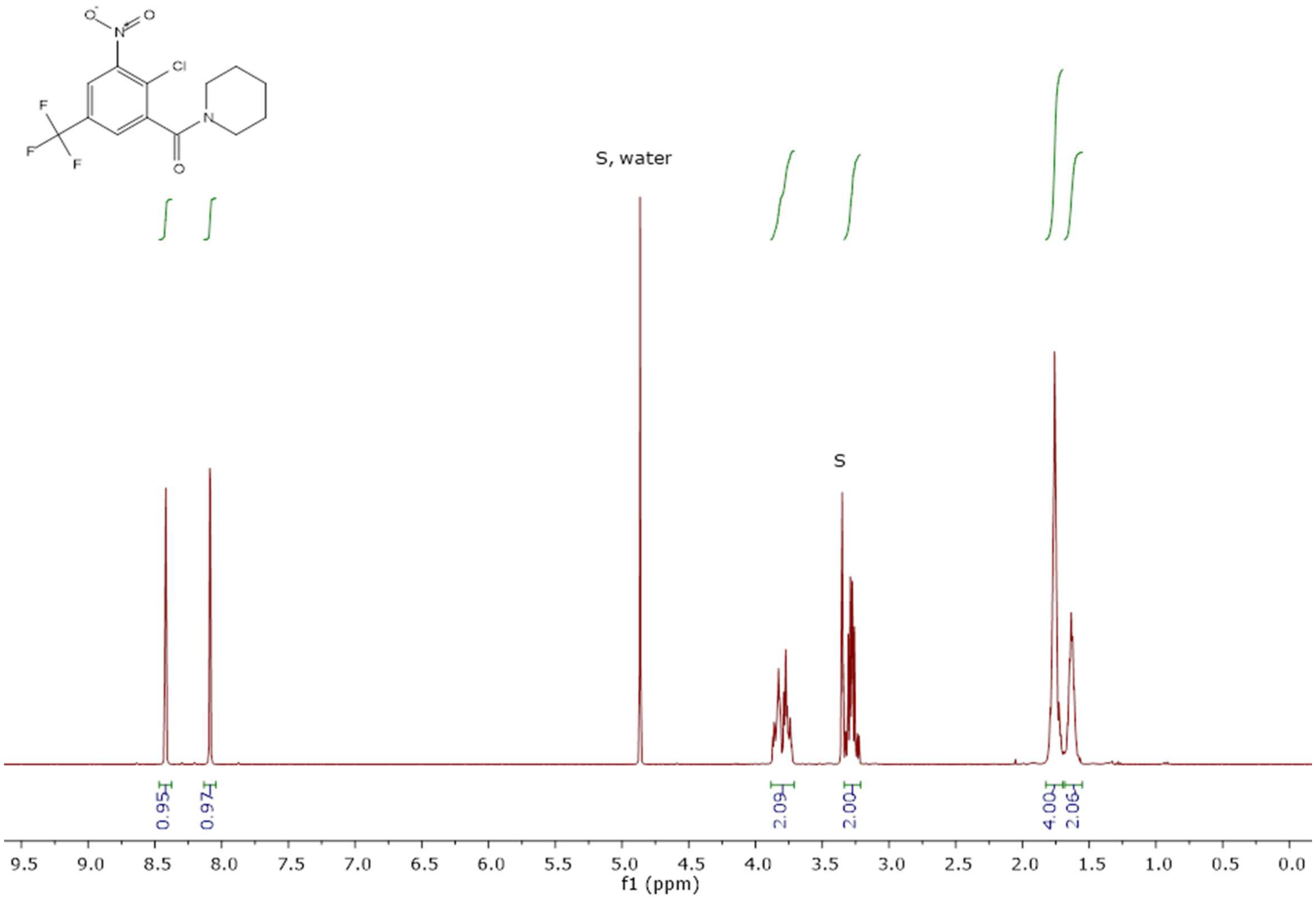

Figure S2 ^{1}H NMR spectrum of **4** in methanol-d_4 at room temperature. S denotes residual solvent signals.

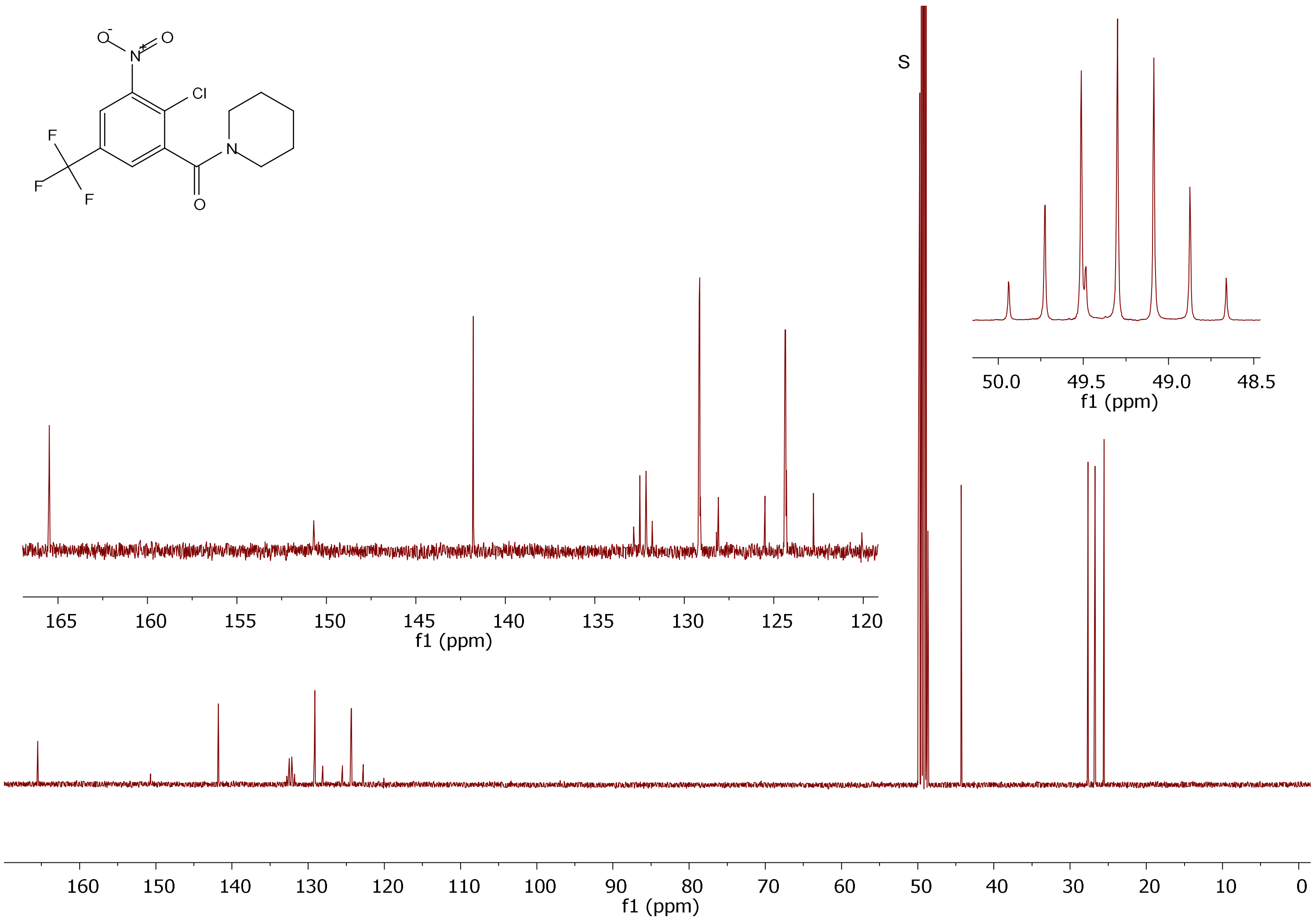

Figure S3 $^{13}C\{^{1}H\}$ NMR spectrum of **4** in methanol-*d*$_4$ at room temperature. S denotes the residual solvent signal. The insets show the aromatic region and the residual solvent septet overlapping with an aliphatic signal of **4** at 49.5 ppm.

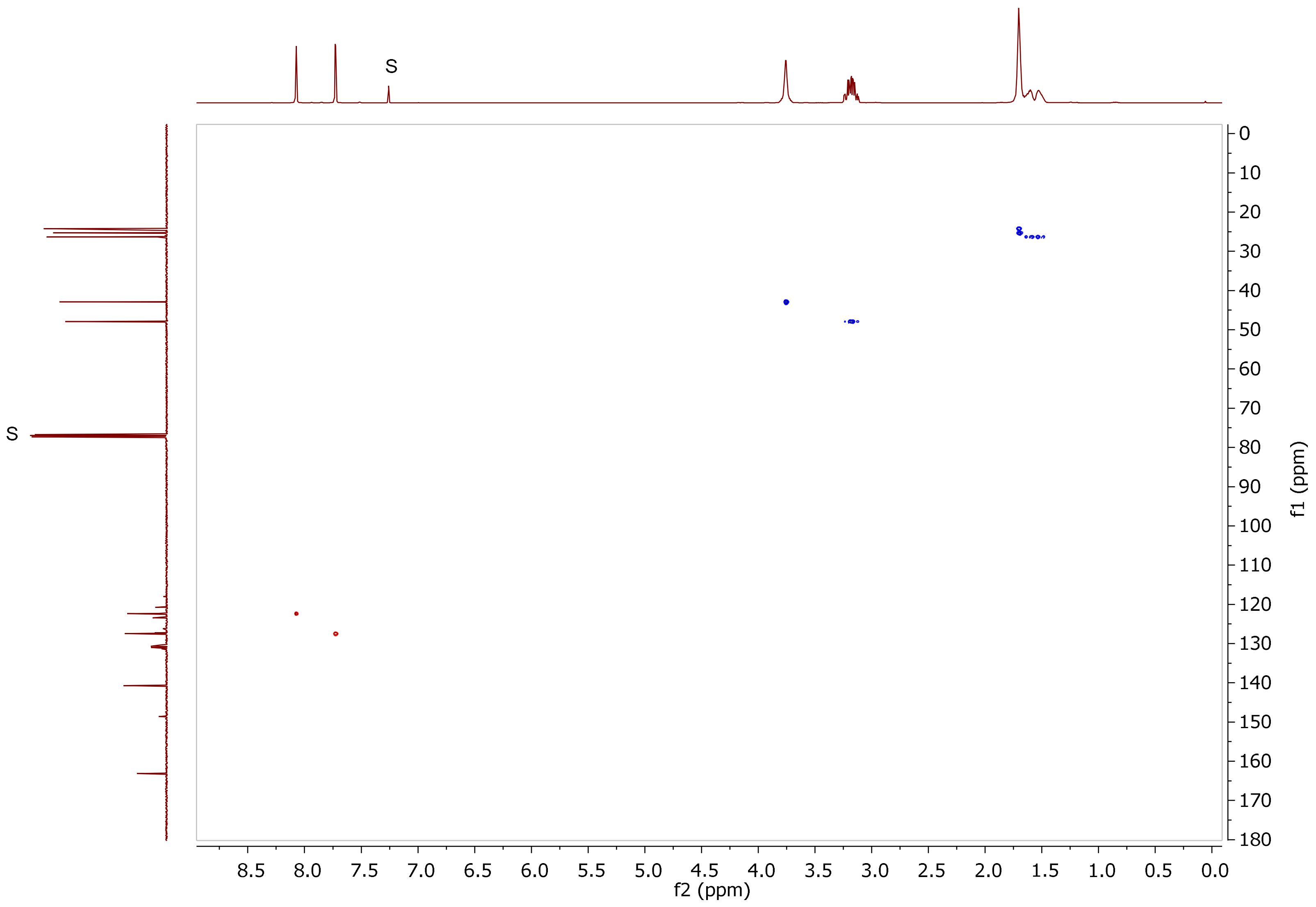

Figure S4 ^{13}C,^{1}H-HSQC NMR spectrum of **4** in chloroform-*d* at room temperature (full spectrum). S denotes the residual solvent signals.

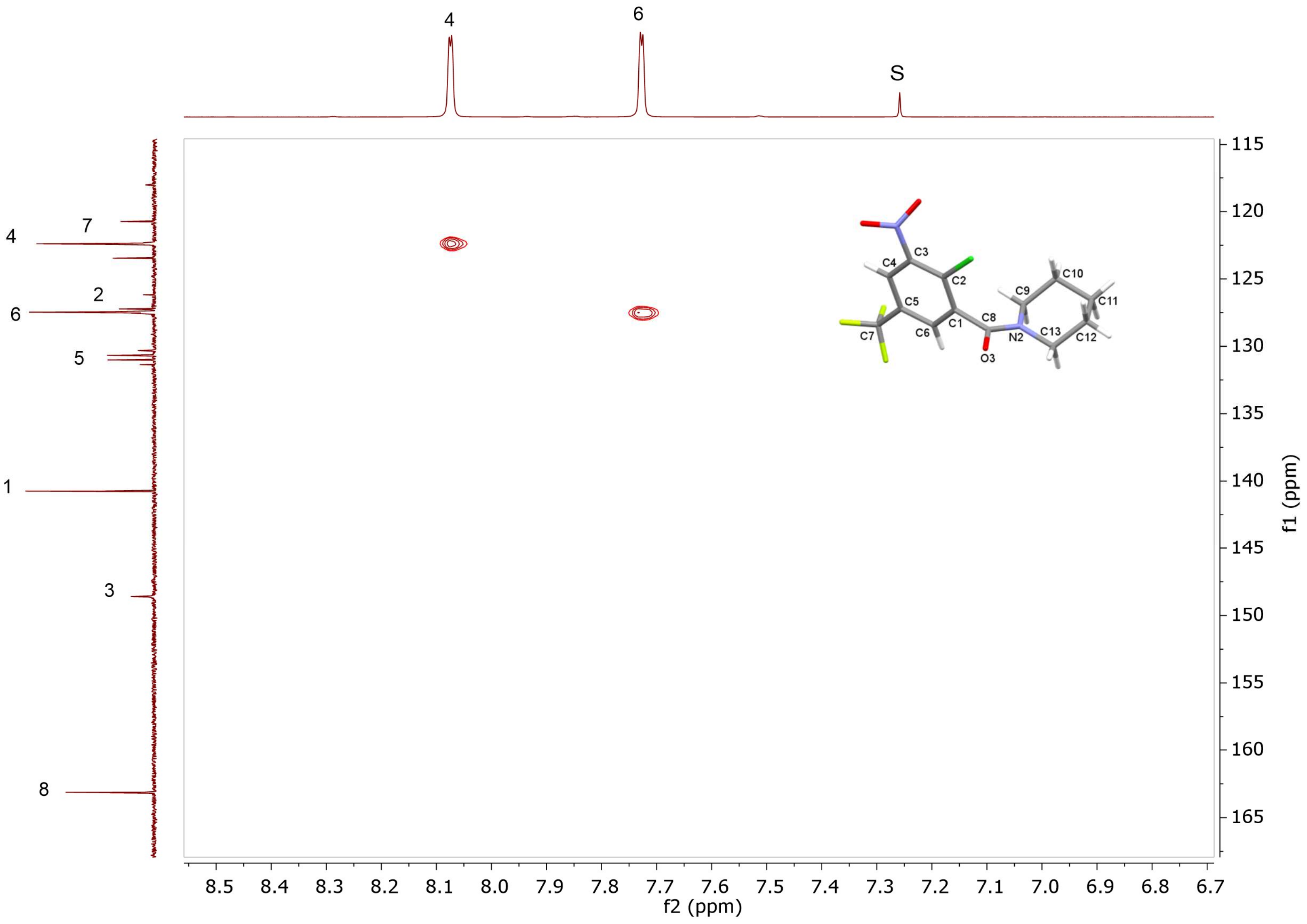

Figure S5 $^{13}C,^{1}H$-HSQC NMR spectrum of **4** in chloroform-*d* at room temperature (aromatic region). S denotes the residual solvent signal.

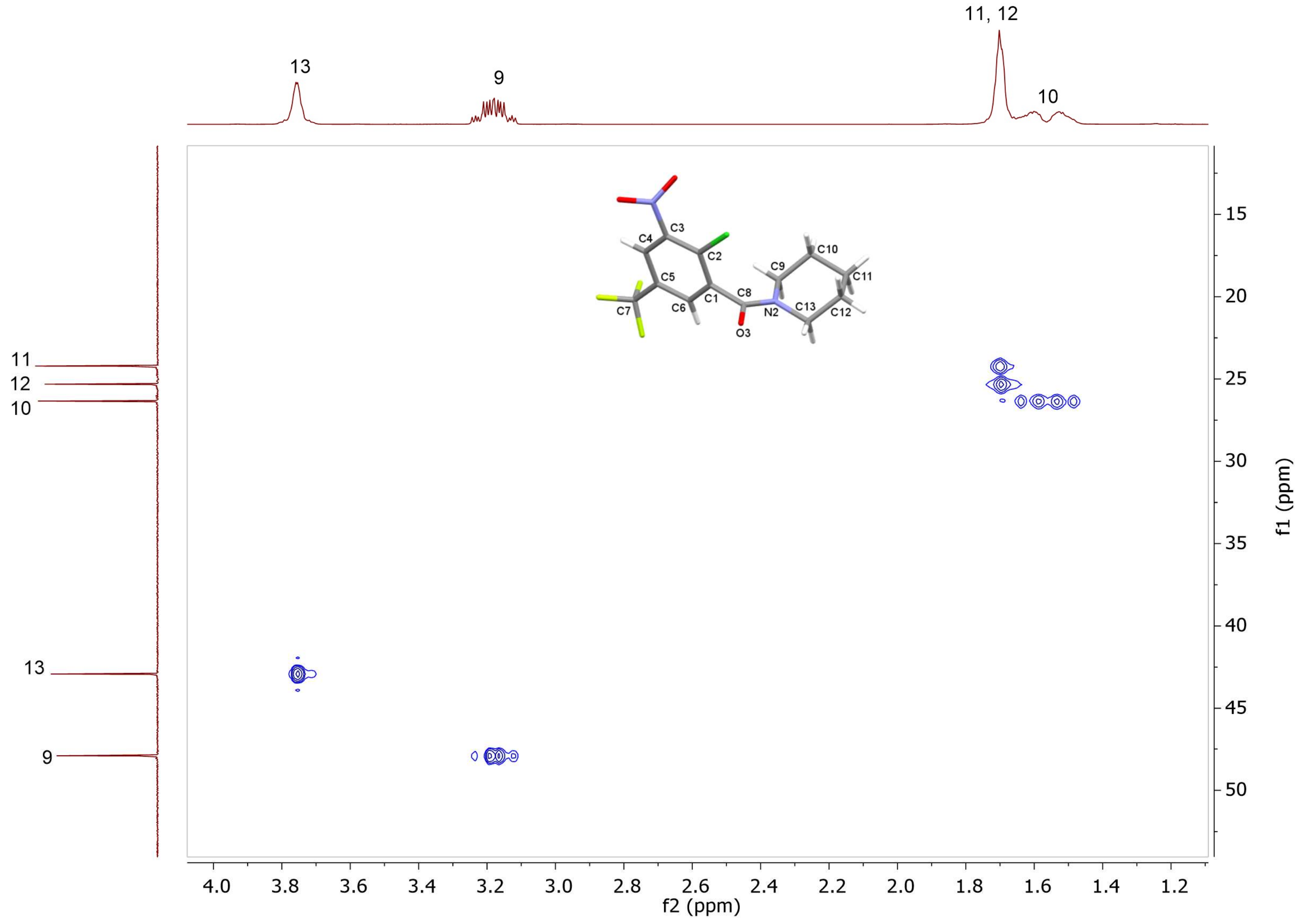

Figure S6 $^{13}C,^{1}H$-HSQC NMR spectrum of **4** in chloroform-*d* at room temperature (aliphatic region).

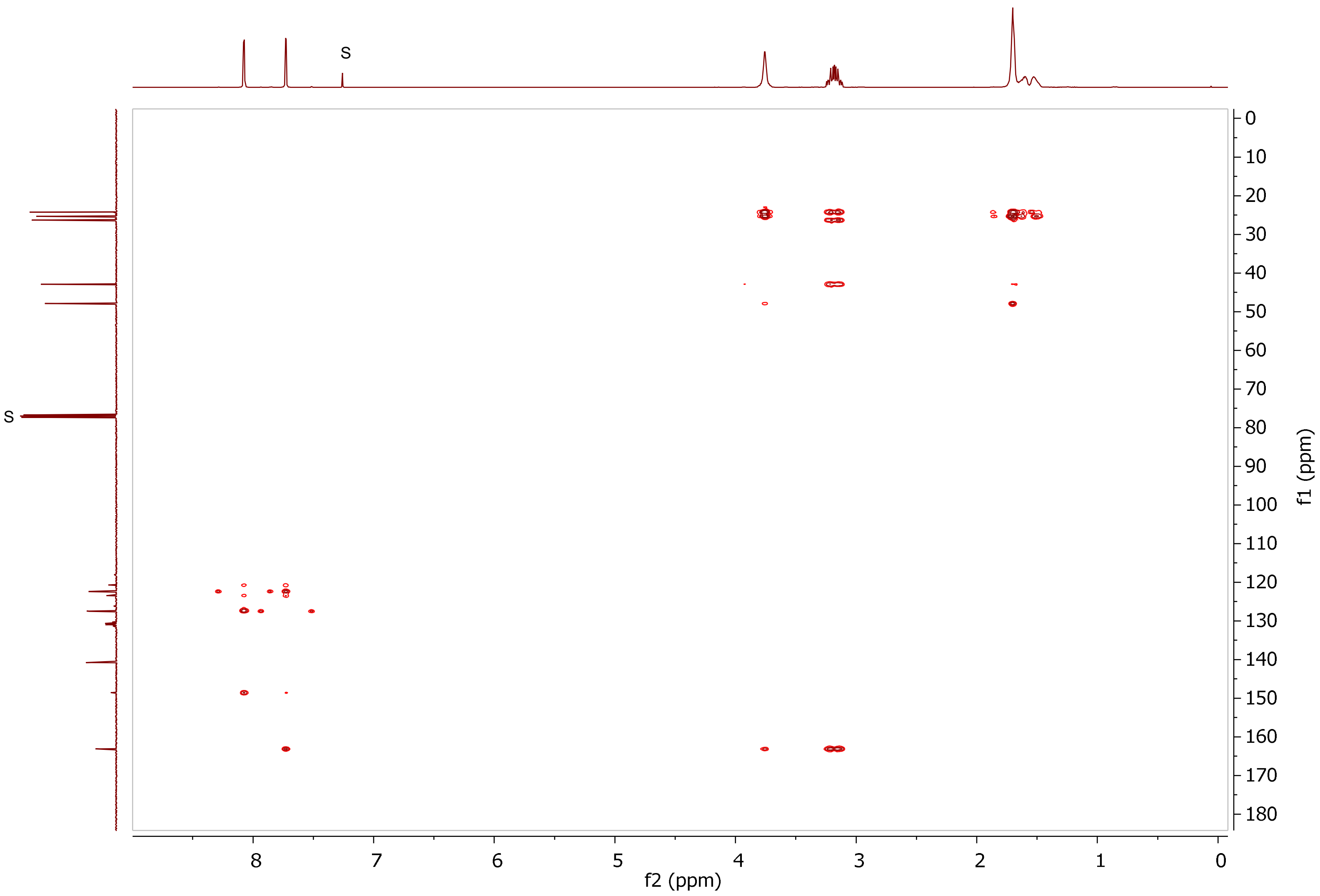

Figure S7 ^{13}C,^{1}H-HMBC NMR spectrum of **4** in chloroform-*d* at room temperature (full spectrum). S denotes the residual solvent signals.

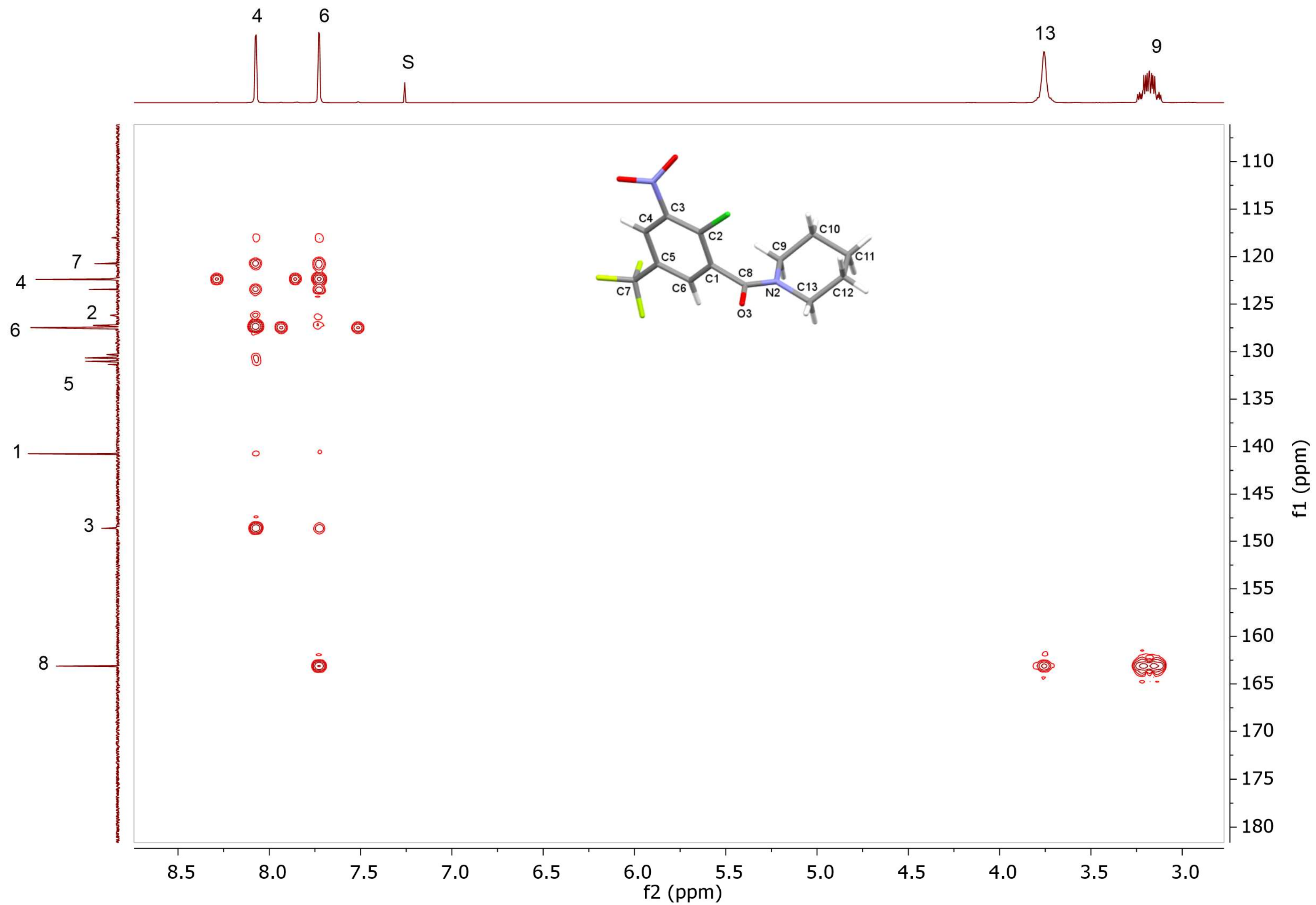

Figure S8 $^{13}C,^{1}H$-HMBC NMR spectrum of **4** in chloroform-*d* at room temperature (aromatic region). S denotes the residual solvent signal.

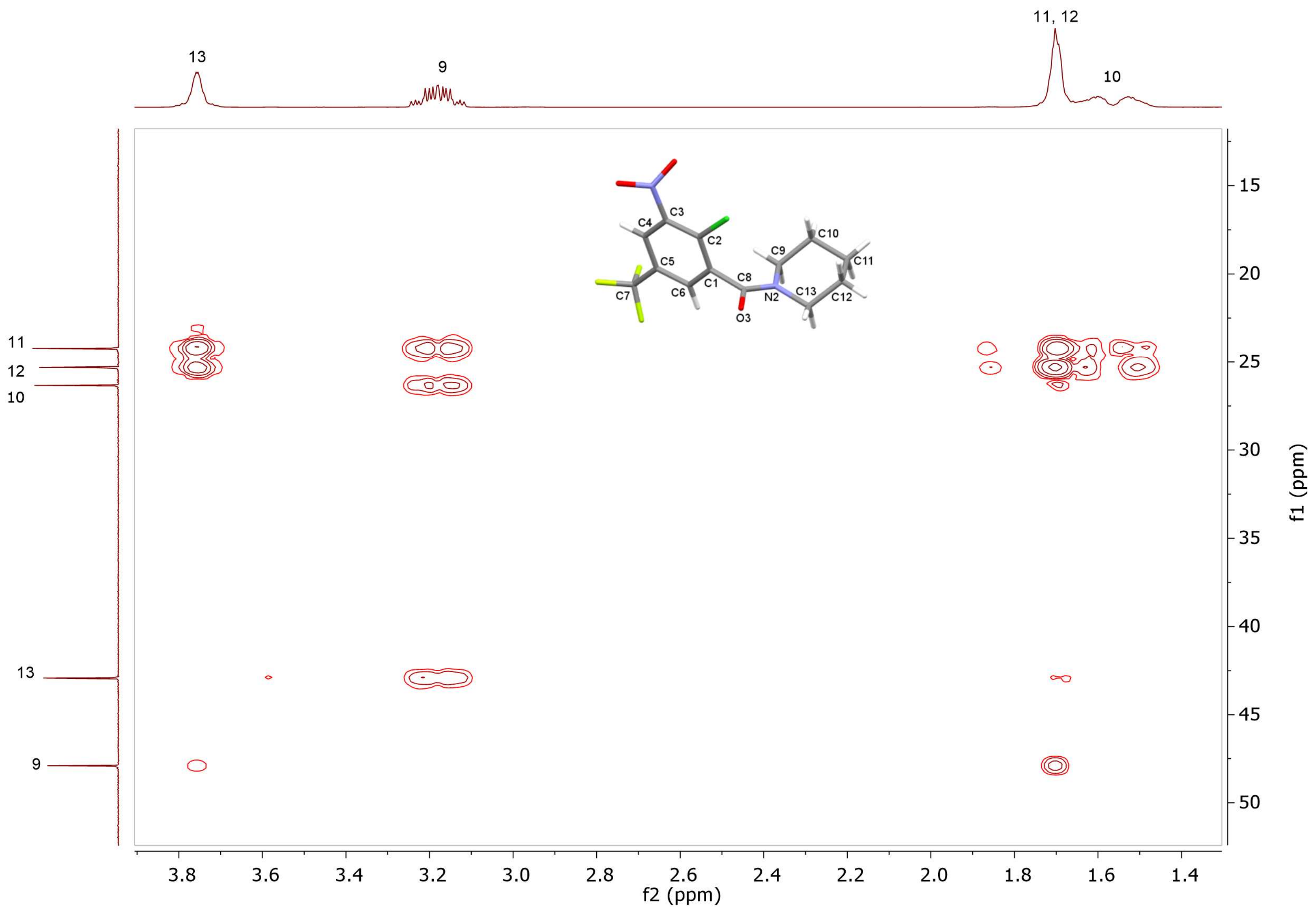

Figure S9 ^{13}C,^{1}H-HMBC NMR spectrum of **4** in chloroform-*d* at room temperature (aromatic region).

MIC determination against *M. smegmatis* mc² 155 pTEC27 and *M. abscessus* ATCC 19977 pTEC27

MICs were determined by the broth microdilution method. 96-well flat bottom tissue culture plates (Sarstedt, 83.3924.500) were used. In the second well of each row, two times the desired highest concentration of each compound was added in 7H9 medium supplemented with 10 % ADS (albumin-dextrose-saline) and 0.05 % polysorbate 80. Each compound was diluted twofold in a 10-point serial dilution.

The concentration of the starting inoculum was 5×10^5 cells/mL. The starting inoculum was diluted from a preculture at the mid-log phase (OD_{600} 0.3 to 0.7) and a OD_{600} of 0.1 was correlated to 1×10^8 CFU/mL. The plates were sealed with parafilm, put in a container with moist tissue and incubated for three days at 37 °C. Each plate had eight negative controls (1 % DMSO) and eight positive controls (100 µM amikacin). After incubation, the plates were monitored by OD measurement at 590 nm (Tecan SpectraFluor). The assay was performed in duplicate and results were validated by RFP measurement.

Data analysis: Every assay plate contained eight wells with DMSO (1 %) as negative control, which corresponds to 100 % bacterial growth and eight wells with amikacin (100 µM) as positive control in which 100 % inhibition of bacterial growth was reached. Controls were used to monitor the assay quality through determination of the Z′ score, which was 0.53 (+/- 0.11). The Z′ factor was determined using the formula (Zhang *et al.*, 1999):

$$Z' = 1 - \frac{3(\mathrm{SD}_{\mathrm{amikacin}} + \mathrm{SD}_{\mathrm{DMSO}})}{(\mathrm{M}_{\mathrm{amikacin}} - \mathrm{M}_{\mathrm{DMSO}})}$$

(SD = standard deviation, M = mean)

The percentage of inhibition was calculated as follows:

$$\%\ \text{inhibition} = (-100) \times \frac{(\mathrm{signal}_{\mathrm{sample}} - \mathrm{signal}_{\mathrm{DMSO}})}{(\mathrm{signal}_{\mathrm{DMSO}} - \mathrm{signal}_{\mathrm{amikacin}})}$$

Zhang, J.H., Chung, T.D. & Oldenburg, K. R. (1999). A simple statistical parameter for use in evaluation and validation of high throughput screening assays. *J. Biomol. Screen.* **4**, 67–73. https://doi.org/10.1177/108705719900400206.
